# Supplementary material for: Uncovering the Genetic Architecture of Optic Nerve Integrity Estimates through Genome-wide Association Study Meta-analyses
Source: medRxiv. 2026 Feb 6:2026.02.03.26345426. Preprint. [Version 1] doi: 10.64898/2026.02.03.26345426 (PMC12889747; doi:10.64898/2026.02.03.26345426)

Uncovering the Genetic Architecture of Optic Nerve Integrity Estimates through Genome-wide Association Study Meta-analyses

Supplementary Figure 1. Q-Q plot comparing the observed (x-axis) versus expected $-\log_{10}$(p-values) (y-axis) under the null hypothesis of no association.

(a) Global pRNFL thickness

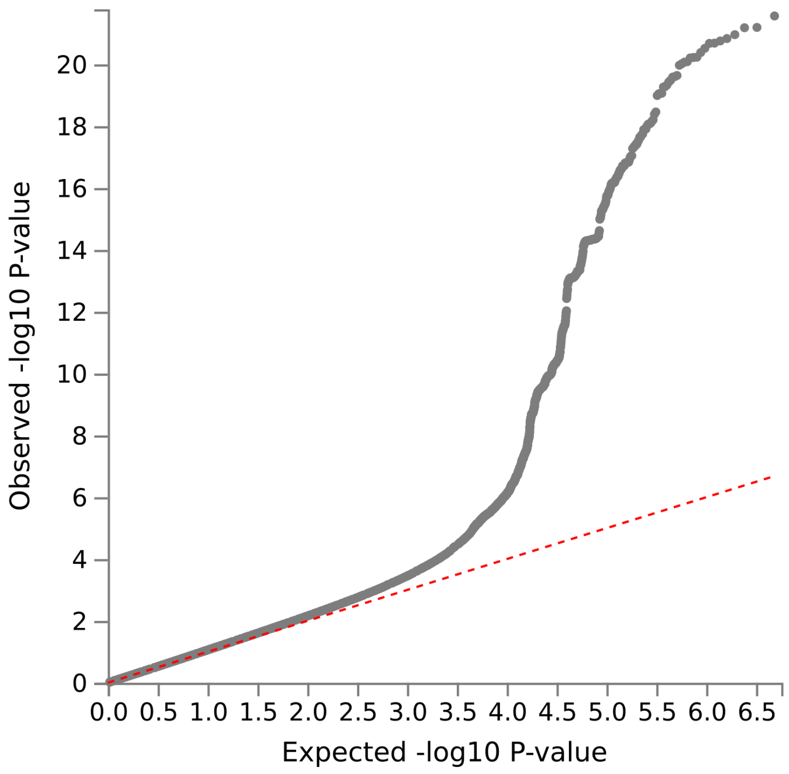

- Lambda GC: 1.0957
- Mean Chi^2: 1.12
- Intercept: 1.0304 (0.0075)
- Attenuation ratio: 0.2536 (0.0622)

(b) Global BMO-MRW

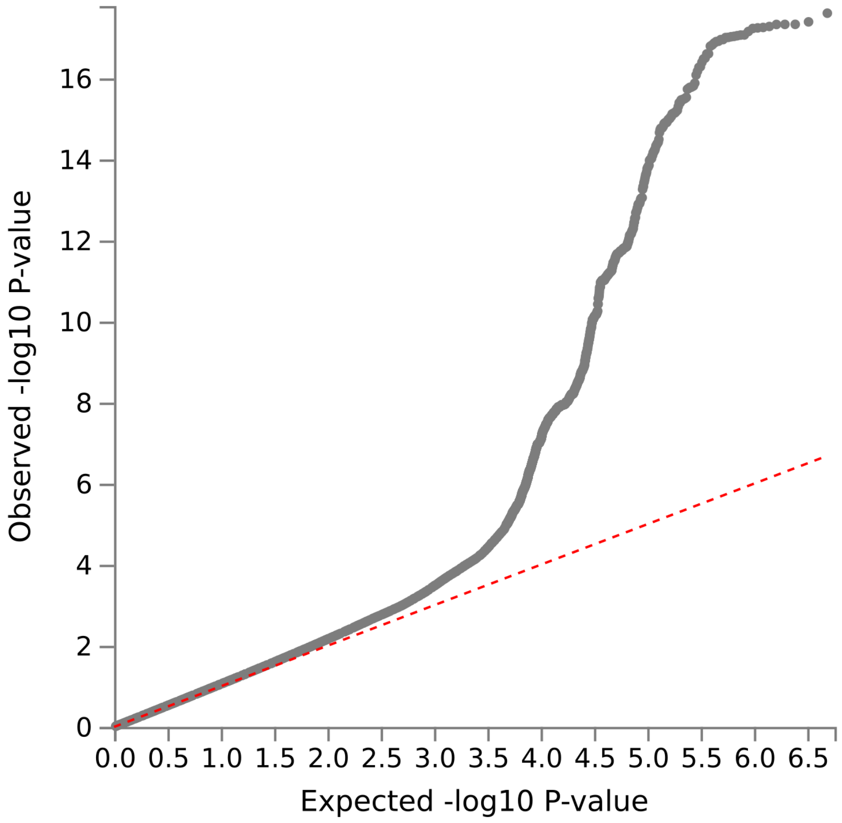

- Lambda GC: 1.0957
- Mean Chi^2: 1.117
- Intercept: 1.0238 (0.0069)
- Attenuation ratio: 0.2038 (0.0587)

Supplementary Figure 2. GWAS meta-analysis results for (a) sectoral pRNFL, (b) sectoral BMO-MRW, and (c) BMO area. Manhattan plot showing the $-\log_{10}$ (p-values) of association tests in the x-axis. Each point represents an SNP plotted by chromosomal position. The horizontal dashed red line indicates the genome-wide significance threshold (p-value = 5×10^{-8}).

(a) Sectoral pRNFL

Temporal	Nasal
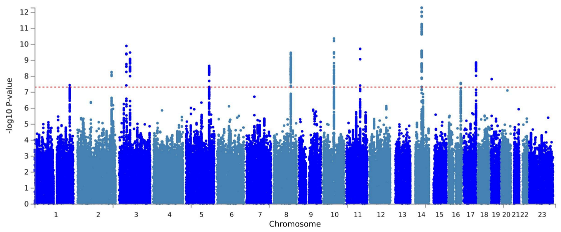	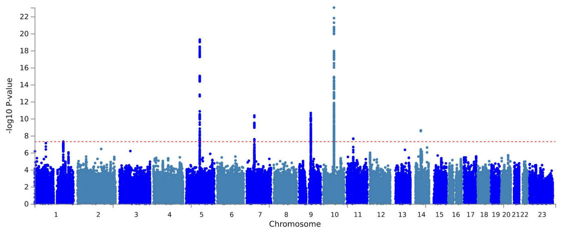
Superotemporal	Superonasal
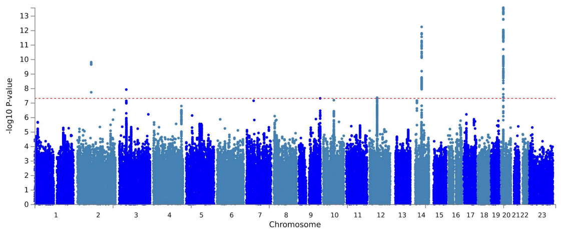	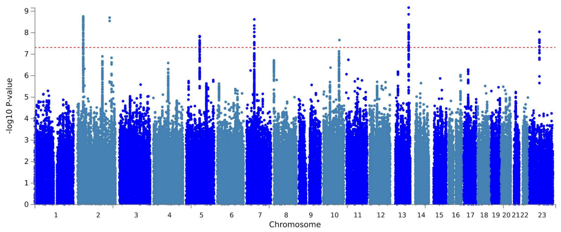
Inferotemporal	Inferonasal
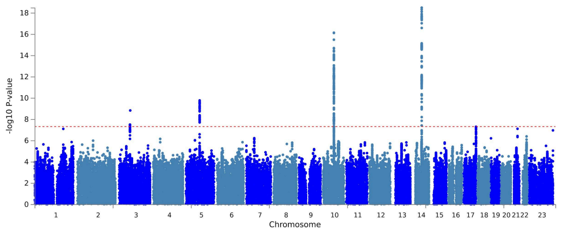	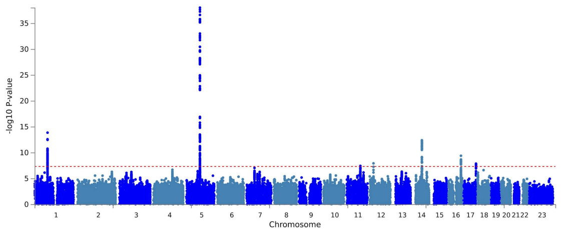

(b) Sectoral BMO-MRW

Temporal	Nasal
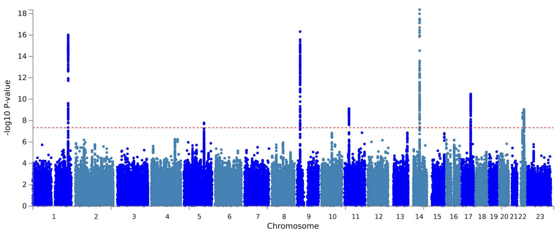	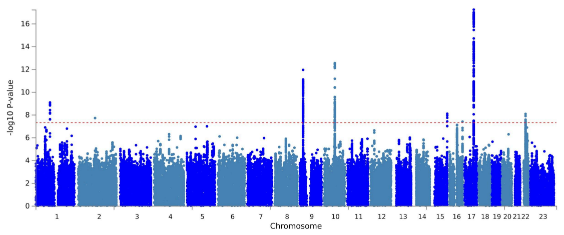
**Superotemporal**	**Superonasal**
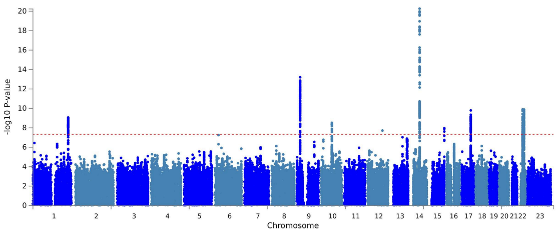	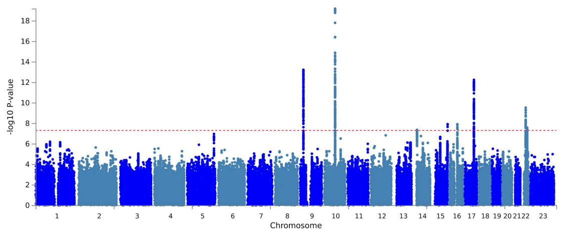
**Inferotemporal**	**Inferonasal**
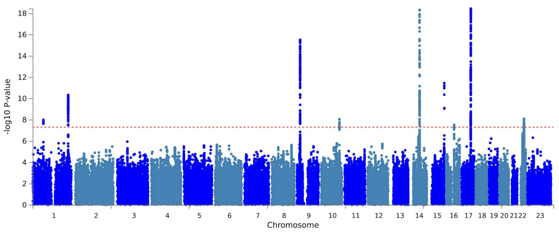	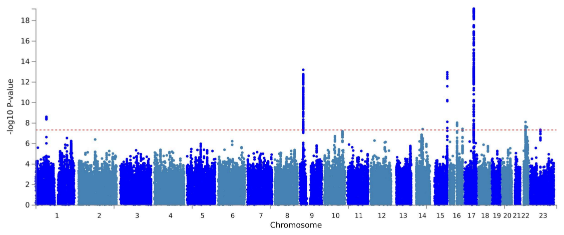

(c) BMO area

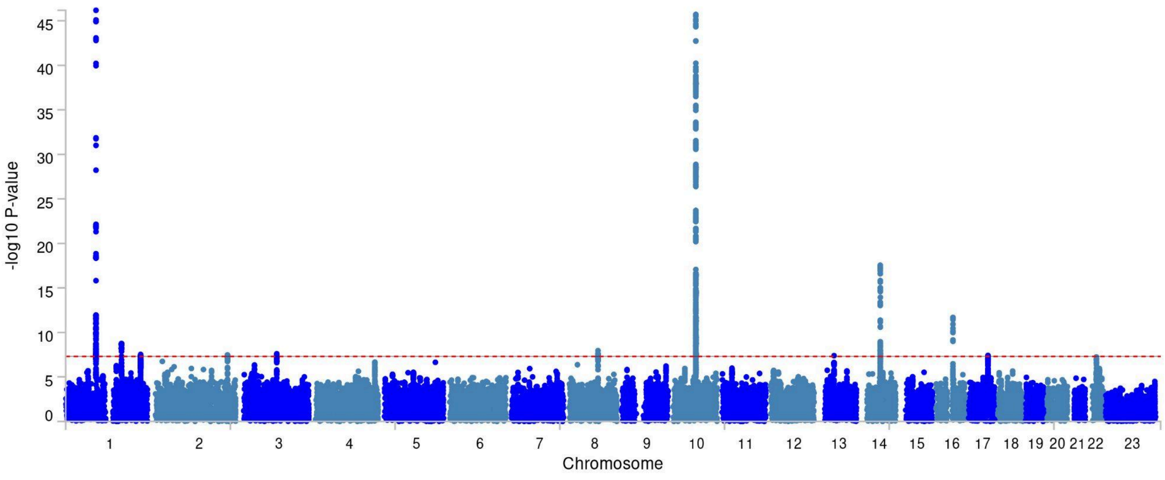

Supplementary Figure 3. Conditional analysis results using the mtCOJO method. Manhattan plots provided for (a) IOP-adjusted global pRNFL thickness and (b) IOP-adjusted global BMO-MRW.

(a) IOP-adjusted global pRNFL thickness

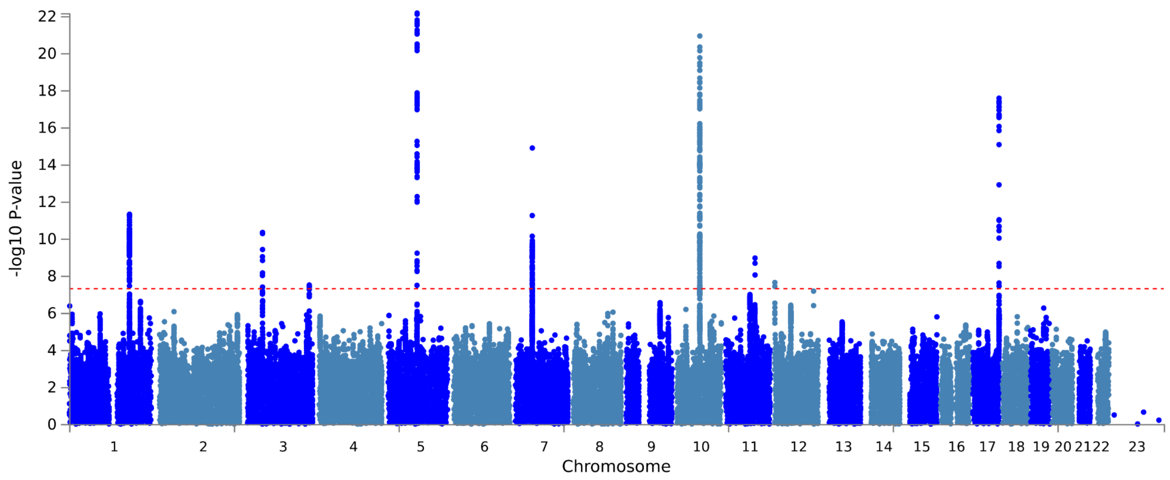

(b) IOP-adjusted global BMO-MRW

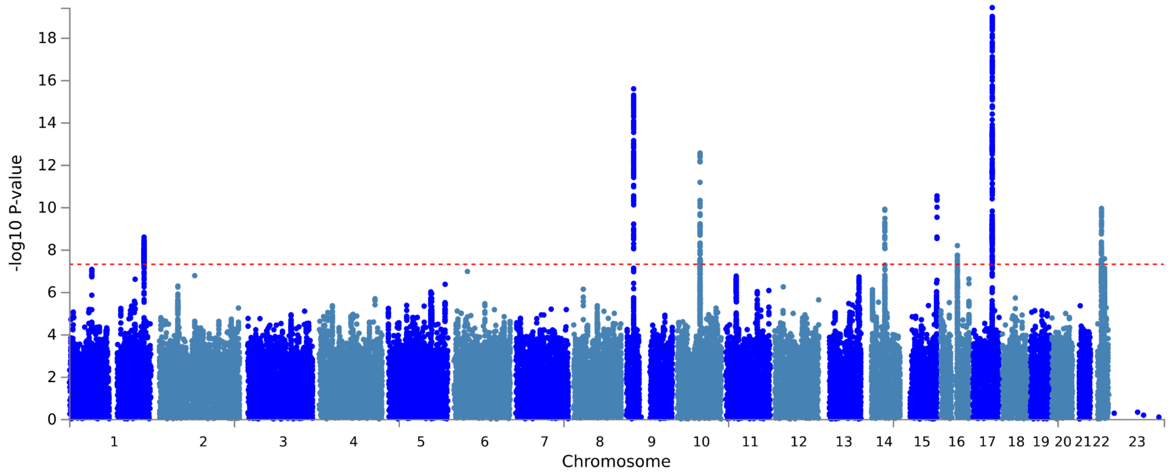

Supplementary Figure 4. Mendelian randomisation scatter plots showing the effects of instrumental variables on the exposure (x-axis) and the outcome (y-axis) across different methods.

(a) IOP on global pRNFL thickness

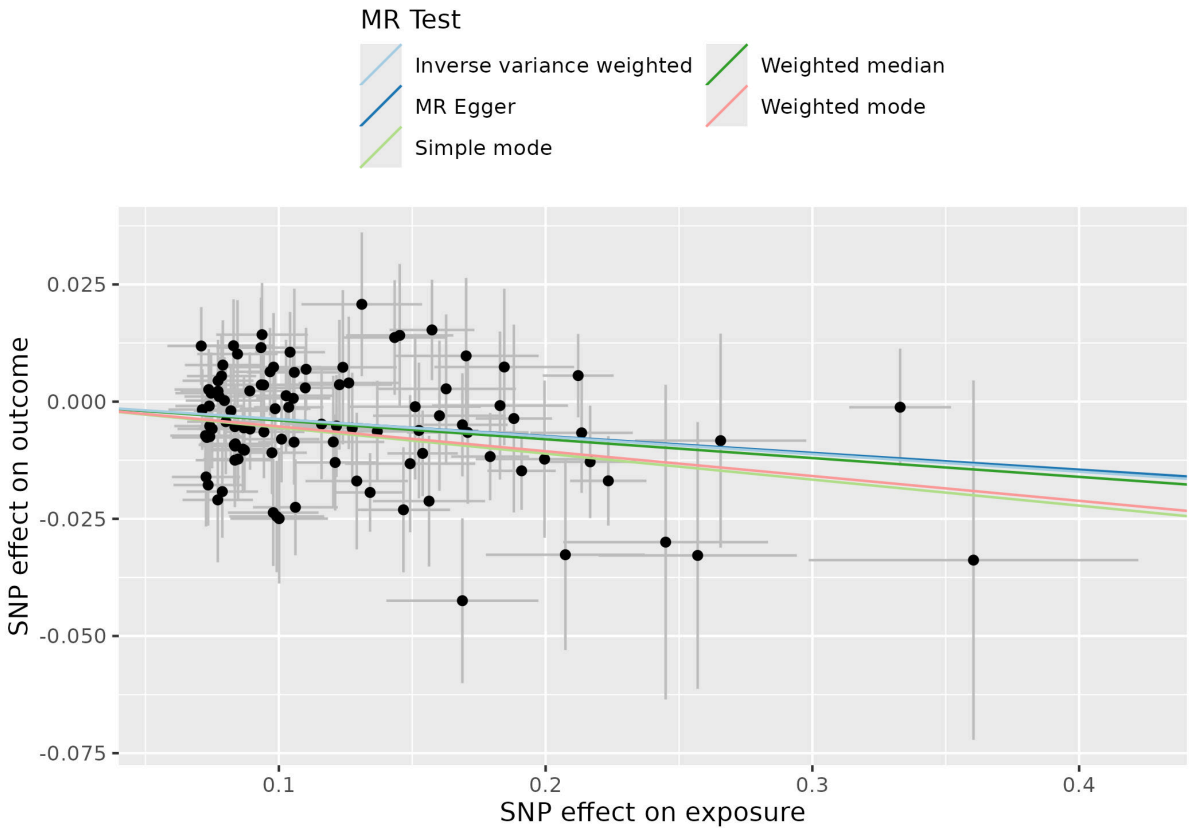

(b) IOP on global BMO-MRW

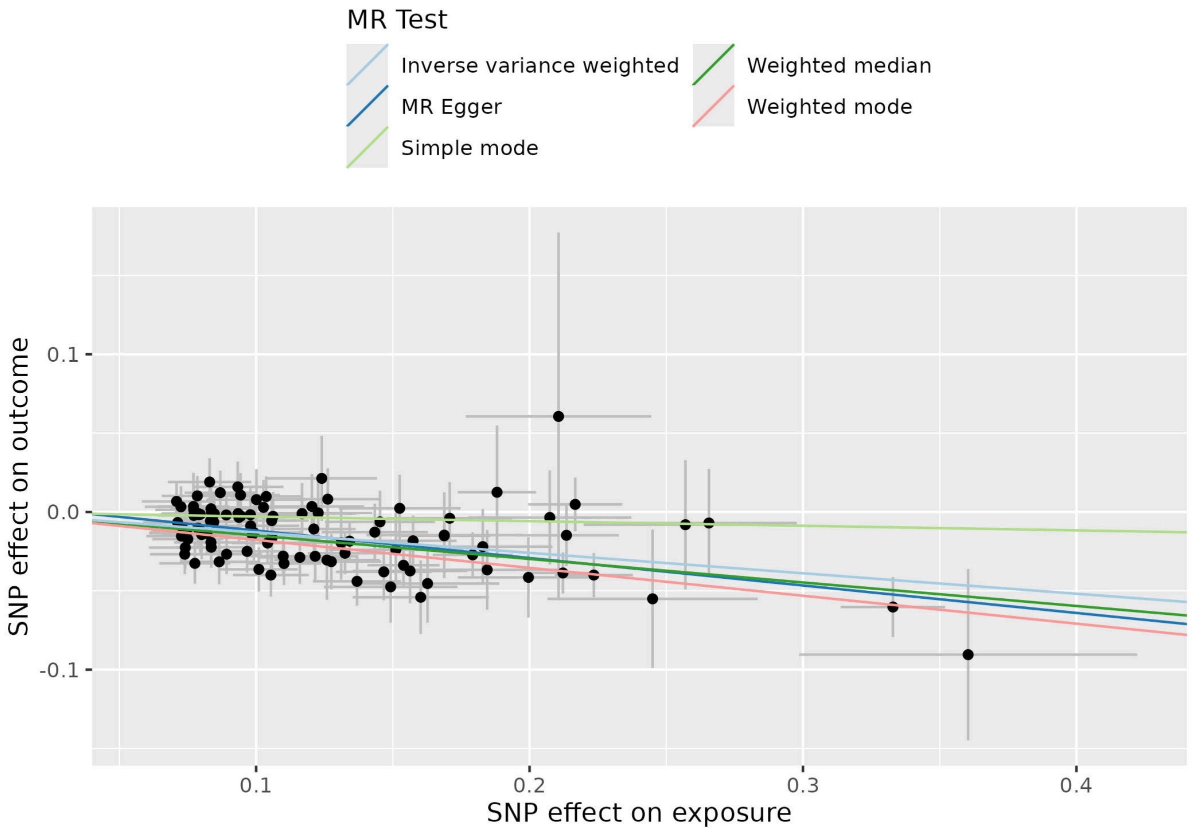

Supplement: Supplementary file 1 [file media-1.pdf]
